# Conversations and Misconceptions About Chemotherapy in Arabic Tweets: Content Analysis

**DOI:** 10.2196/13979

**Published:** 2020-07-29

**Authors:** Abdulrahman Alghamdi, Khalid Abumelha, Jawad Allarakia, Ahmed Al-Shehri

**Affiliations:** 1 College of Medicine King Saud Bin Abdulaziz University for Health Sciences Jeddah Saudi Arabia; 2 King Abdullah International Medical Research Center Jeddah Saudi Arabia; 3 Department of Medical Oncology Princess Noorah Oncology Center Ministry of the National Guard - Health Affairs Jeddah Saudi Arabia

**Keywords:** internet, chemotherapy, cancer, Twitter, social media, Arab world, misconceptions, infodemiology, infoveillance

## Abstract

**Background:**

Although chemotherapy was first introduced for the treatment of cancer more than 60 years ago, the public understanding and acceptance of chemotherapy is still debatable. To the best of our knowledge, no study has assessed the conversations and misconceptions about chemotherapy as a treatment for cancer on social media platforms among the Arabic-speaking populations.

**Objective:**

The aim of this study was to assess the types of conversations and misconceptions that were shared on Twitter regarding chemotherapy as a treatment for cancer among the Arabic-speaking populations.

**Methods:**

All Arabic tweets containing any of the representative set of keywords related to chemotherapy and written between May 1, 2017 and October 31, 2017 were retrieved. A manual content analysis was performed to identify the categories of the users, general themes of the tweets, and the common misconceptions about chemotherapy. A chi-square test for independence with adjusted residuals was used to assess the significant associations between the categories of the users and the themes of the tweets.

**Results:**

A total of 402,157 tweets were retrieved, of which, we excluded 309,602 retweets and 62,651 irrelevant tweets. Therefore, 29,904 tweets were included in the final analysis. The majority of the tweets were posted by general users (25,774/29,904, 86.2%), followed by the relatives and friends of patients with cancer (1913/29,904, 6.4%). The tweets were classified into 9 themes; prayers and wishes for the well-being of patients undergoing chemotherapy was the most common theme (20,288/29,904, 67.8%), followed by misconceptions about chemotherapy (2084/29,904, 7.0%). There was a highly significant association between the category of the users and the themes of the tweets (χ^2^_40_= 16904.4, *P*<.001).

**Conclusions:**

Our findings support those of the previous infodemiology studies that Twitter is a valuable social media platform for assessing public conversations, discussions, and misconceptions about various health-related topics. The most prevalent theme of the tweets in our sample population was supportive messages for the patients undergoing chemotherapy, thereby suggesting that Twitter could play a role as a support mechanism for such patients. The second most prevalent theme of the tweets in our study was the various misconceptions about chemotherapy. The findings of our exploratory analysis can help physicians and health care organizations tailor educational efforts in the future to address different misconceptions about chemotherapy, thereby leading to increased public acceptance of chemotherapy as a suitable mode of treatment for cancer.

## Introduction

Although chemotherapy linguistically refers to any type of treatment with chemicals, it is now specifically used to describe cytotoxic anticancer medications, which are used to treat hematological and solid tumors [1,2]. Chemotherapeutic agents can be used alone or in combination with surgery or radiotherapy to achieve the goal of the treatment, which varies among patients based on many factors. The goal of the treatment may be to cure the cancer completely, control the cancer, stop it from spreading, or to use these agents as a palliative means to ease the symptoms and improve the patients’ quality of life [3]. However, the natural mechanism of action of these chemotherapeutic agents results in relatively a wide spectrum of side effects, which might be unpleasant to the patients.

Despite continuous improvements in chemotherapy for increasing its effectiveness in treating cancer, the public understanding and acceptance of chemotherapy is still debatable. Many misconceptions can occur, thereby causing compliance issues and other problems related to the acceptance of chemotherapy as a treatment modality [4]. Most of the previous studies that assessed the perceptions and experiences of chemotherapy were performed with selected groups of patients who were either surveyed or interviewed [5-8]. However, large-scale studies attempting to understand the public views and conversations on chemotherapy are lacking. Retrieving and analyzing the contents of various social media platforms on this matter serves as an evolving method to gather information about the perceptions, misconceptions, and experiences with chemotherapy as a treatment for cancer. One of the most popular social media platforms that has been explored is Twitter, which is a large microblog wherein users can write up to 280-character messages called tweets and share them with their followers and the public. There are almost 313 million monthly active users who write around 500 million tweets daily [9,10]. In March 2017, it was estimated that the number of the monthly active Twitter users in the Arab world was around 11.1 million [11]. Moreover, the number of their tweets per month in March 2016 was estimated to be 849.1 million tweets, and 72% of them were written in Arabic [11].

Twitter can be useful for exploring public opinions on health-related matters and it may play an important role in clinical settings by providing an avenue for patients to increase their knowledge regarding their diseases and by leading to a positive impact on their medical outcomes [12-14]. For example, a study showed that the mental health of the patients was affected by social media content, as positive messages were shown to reduce anxiety in patients with breast cancer who were using Twitter [12]. Moreover, social media contents show the public impression and knowledge in addition to their emotions and stress regarding diseases and their associated factors in real time [15,16].

The aim of this study was to report a content analysis of the Arabic tweets regarding chemotherapy to determine the categories of the contents and to explore the possible cases of misconceptions about chemotherapy in the Arab world. This study addresses the common areas of misconceptions on cytotoxic chemotherapies in Arabic Twitter conversations, which may help to direct the future educational efforts to address these areas of misconception.

## Methods

### Data Collection

This observational study was conducted on Arabic tweets about chemotherapy over a 6-month period, and the main themes to which these tweets belonged were determined. A search was conducted on every publicly available tweet in Arabic containing any of the keywords mentioned in the supplementary file (Multimedia Appendix 1). Keywords were chosen on a linguistic basis; therefore, they represented all the possible spellings and abbreviations of the word “chemotherapy” in Arabic. The Twitter Archiver add-on to Google Sheets was used to collect the data [17]. This tool searches for new public tweets every 15 minutes that include any prespecified keywords and allows users to download them on a Microsoft Excel spreadsheet. Further, this tool collects the related data of every user whose tweet has been collected such as the number of people who follow those users, whom they follow, and a brief biography that users may share about themselves on the profile page. All Arabic tweets from May 1, 2017 to October 31, 2017 that contained any of the predetermined keyword(s) were retrieved. Among these, we excluded all retweets because they might deviate from the thematic analysis as they do not necessarily imply endorsement and we excluded irrelevant tweets containing the keyword but that were used in a context other than chemotherapy as a medical intervention (eg, chemical weapon attacks in Syria).

### Data Analysis

To help expand the knowledge on the tweets circulated on chemotherapy, the main questions asked to generate the coding scheme were if there were misconceptions or not about chemotherapeutic agents as cancer treatments and what other patterned meanings appeared in the data set. After a thorough study of a sample of 150 tweets, the primary author generated a preliminary list of the themes of the tweets and the categories of the users. This list was used by the rest of the authors to code the same 150-tweet sample for the themes and the categories of the users, which resulted in the modification and addition of a few themes. Thereafter, another 150 tweets were used to finally test the comprehensiveness of the themes and the categories of the users along with assessing the inter-rater reliability of the coders of the main data set (JA and KA) by using the Cohen kappa coefficient. The Cohen kappa coefficients were estimated as 0.90 and 0.92 for themes and categories of users, respectively. Table 1 describes the themes of the tweets and the illustrative quotes translated from Arabic. Table 2 describes the different categories of the users who were identified on the basis of the information provided in their biographies mentioned in their Twitter profile page or on the basis of their tweets.

Using the coding scheme, 2 of the investigators (JA and KA) independently assigned each tweet and the user who shared it to the proper mutually exclusive theme and user category. Cases of ambiguity were resolved by consensus among the authors. Tweets under the misconception theme were further analyzed to establish the most common misconceptions among the studied groups of users.

**Table 1:** Descriptions of the different themes of the tweets on chemotherapy and the illustrative tweets.

Theme of the tweet	Description	Examples of the translated tweets
Advice and information	Disseminating true information and advice about chemotherapy	…*We use chemotherapy because its efficacy is proven—that is why it is called as evidence-based medicine*.
Experience	Sharing an experience of chemotherapy either by the patients themselves or by the people who surround them	…*My brother is on chemotherapy—the first session was on his birthday. It was like a birthday present—a painful and sad present.*
Misconception	Sharing a false concept or a false idea about chemotherapy	…*a long time ago I heard that a cure for cancer was discovered, but the person who discovered it was killed because companies wanted to benefit from the profits that are generated from selling chemotherapeutic drugs.*
Prayers and wishes	Saying a prayer or a wish for recovery for people receiving chemotherapy	…*They have been worn out by cancer, by chemotherapy…oh Allah heal our patients and all Muslim patients.*
Seeking medical information/advice	Asking for medical information about chemotherapy	…*If a patient has started chemotherapy for treating cancer, does he lose only the scalp hair or does he lose the hair all over his body?*
Seeking medical/financial help	Asking for medical or financial help for patients receiving chemotherapy	…*My mother is supposed to receive chemotherapy this week but we cannot afford it. All radiographic images and reports are attached. We need your help.*
Offering medical intervention/financial help	Offering medical or financial help for patients needing or receiving chemotherapy.	…*If anyone is in need of paclitaxel, which is a chemotherapeutic drug that is well over 3000 Egyptian pounds (US $187), please do not hesitate to contact me.*
Analogy	Using chemotherapy in an analogic way to share an idea or a concept.	…*Whatever concerns you now is nothing compared to those who are waiting for chemotherapy or hemodialysis tomorrow.*
Miscellaneous	Tweets that did not fit any of the other categories.	…*I hope I can discover a cancer treatment better than chemotherapy.*

**Table 2:** Description of the categories of the different Twitter users who tweeted on chemotherapy.

Category of the Twitter user accounts	Description of the Twitter user accounts
Patients with cancer/survivors	Accounts of patients with cancer receiving chemotherapy
Relatives/friends of patients with cancer	Accounts of a relative or a friend of a patient receiving chemotherapy
Cancer specialists	Accounts of people working in the oncology field
Health-related accounts	Accounts of an organization in the medical field or of a person working in a medical field other than oncology
Media-related accounts	Accounts of media platforms such as newspapers and news channels
General users	Accounts of anyone who does not belong to the other categories or who belongs to unidentified accounts.

### Statistical Analysis

Statistical analysis was performed using the SPSS software (release version 23.0.0.0, IBM Corp). Descriptive statistical analyses were used to report the frequencies for each theme and the category of the users. The chi-square test for independence was used to assess any significant associations between the types of users and the themes of the tweets. To further identify the cells that contributed to the overall significant results, adjusted residuals (z-scores) were calculated for each cell along with the *P* values, as described by Beasley and Schumacker [18]. Taking into consideration the multiple comparisons in this analysis that might lead to type I error, a Bonferroni-adjusted *P*<.001 was utilized to indicate statistical significance.

## Results

### Frequency of Themes and Categories of Users

**Figure 1:**
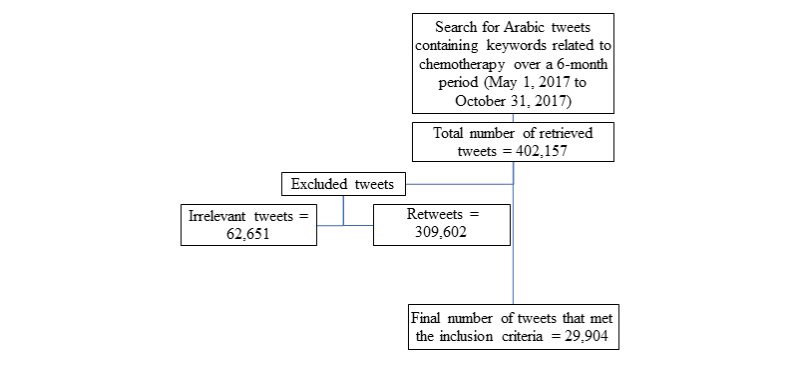
Overview of the process of collecting and filtering the data.

A total of 402,157 tweets were retrieved. Of these, we excluded 309,602 retweets and 62,651 irrelevant tweets (Figure 1). Therefore, 29,904 tweets were included in the final analysis. Table 3 provides the frequencies of all the themes and the user categories. Under the 9 previously identified themes, over two-thirds of the tweets (20,288/29,904; 67.8%) expressed “prayers and wishes” for the recovery of patients with cancer on chemotherapy. Approximately 7.0% (2084/29,904) of the tweets contained misconceptions regarding various aspects of chemotherapy. Providing “advice and information” was the third most frequent theme (1888/29,904, 6.3%), followed by tweets expressing “experience” (1847/29,904, 6.2%) with chemotherapy for cancer management and, lastly, tweets that used 1 or more of the keywords for “analogy” (1556/29,904, 5.2%).

**Table 3:** Frequency analysis of the themes of the tweets on chemotherapy and the Twitter user categories (N=29,904).

Variable		n (%)	
**Themes**			
	Miscellaneous		932 (3.1)
	Advice and information		1888 (6.3)
	Experience		1847 (6.2)
	Misconception		2084 (7.0)
	Prayers and wishes		20,288 (67.8)
	Seeking medical information/advice		487 (1.6)
	Seeking medical/financial help		754 (2.5)
	Offering medical/financial help		66 (0.2)
	Analogy		1556 (5.2)
**Category of users**			
	General users		25,774 (86.2)
	Patients with cancer/survivors		854 (2.9)
	Relatives/friends of patients with cancer		1913 (6.4)
	Cancer specialists		222 (0.7)
	Health-related accounts		459 (1.5)
	Media-related accounts		680 (2.3)

In terms of the proportions of the categories of the users, general users represented the majority of the users in the analyzed data set (25,774/29,904, 86.2%). The relatives and friends of patients with cancer were the second most common contributors (1913/29,904, 6.4%), followed by patients with cancer and survivors (854/29,904, 2.9%). Cancer specialists, health-related accounts, and media-related accounts represented the remaining less than 5% (1361/29,904, 4.5%) of the tweets. Tweets under the theme “misconceptions” were further analyzed to capture the most common misconceptions, as described in detail in Table 4. Falsified and unrealistic side effects of chemotherapy were the most common misconceptions (1271/2084, 60.9%) in the studied data set. The other misconceptions were that chemotherapy causes cancer to spread (282/2084, 13.5%) and that chemotherapy has no therapeutic benefit (214/2084, 10.3%).

We compared the volumes of the tweets of misconception with the volumes of the tweets by cancer specialists and health-related accounts for the same period, and we found that the 2 noticeable spikes on June 1-16, 2017 and July 1-16, 2017 in the volume of the tweets containing misconceptions seemed to drive both categories of users to tweet more (Table 5).

**Table 4:** Frequency of the main misconceptions about chemotherapy in the Arabic tweets (n=2084).

Misconceptions	n (%)
Falsified and unrealistic side effects about chemotherapy; the main shared tweet was that “a drop of chemotherapy spilled on a healthy man’s skin would severely burn the skin.”	1271 (60.9)
Chemotherapy causes cancer to spread.	282 (13.5)
Chemotherapy has no therapeutic effect on cancers.	214 (10.3)
Claims that there are natural products/preparations (eg, olive oil, *Nigella sativa*, ginger, almonds, camel milk or urine) or other practices (eg, blood cupping, carbohydrate restriction), which are more effective than chemotherapy for treating cancers	170 (8.2)
Chemotherapy is prescribed so that pharmaceutical companies and physicians can make huge profits.	67 (3.2)
Claims about some pharmaceutical products (eg, vitamin B17, antibiotics, vitamin C) being more effective than chemotherapy for treating cancers	47 (2.3)
Claims that there are few religious practices (eg, Roqya, Zamzam water intake, seclusion in mosque, fasting from dawn to sunset), which are more effective than chemotherapy for treating cancers	33 (1.6)

**Table 5:** Comparison of the number of tweets containing misconceptions by any user category and the number of tweets by cancer specialists and health-related accounts during the study period.

Time period (2017)	Tweets containing misconceptions (n)	Tweets by cancer specialists and health-related accounts (n)
May 1-16	1616	35
May 17-31	1992	33
June 1-16^a^	4365	108
June 17-30	2561	41
July 1-16^a^	3441	104
July 17-31	2854	84
August 1-16	2486	21
August 17-31	2758	59
September 1-16	2026	49
September 17-30	2670	51
October 1-16	1372	52
October 17-31	1761	44

^a^Noticeable spikes in the number of tweets containing misconceptions and tweets by cancer specialists and health-related accounts.

### Comparison of the Themes by the Categories of the Users

The initial Pearson chi-square test of independence showed a highly significant association between the category of the users and the themes of tweets (*χ^2^*_40_=16904.4, *P*<.001). Further post-hoc analyses revealed the cells that contributed the overall significance (Table 6).

Table 6 shows that general users were more likely to support patients with cancer by tweeting prayers and wishes and less likely to tweet about experiences, seeking medical intervention, and advice and information themes. Patients with cancer and the relatives and friends of patients with cancer tweeted significantly more about experiences and seeking medical advice and intervention than all other themes. They were less likely to tweet misconceptions. Cancer specialists were more likely to tweet advice and information about chemotherapy and less likely to tweet either prayers and wishes or misconceptions. Health-related accounts showed a similar statistically significant tendency as the cancer specialists to tweet more about advice and information, but they had a greater tendency to tweet about offering medical interventions. Finally, media-related accounts were more likely to tweet about advice and information but also more likely to share misconceptions than all others. In contrast, they were less likely to share tweets on prayers and wishes or tweets containing analogy.

**Table 6:** Distribution of the themes by the source category.

Total number of tweets in each user category (n)	Theme-wise tweets								
	Miscellaneous, n (%)	Advice and information n (%)	Experience n (%)	Misconception n (%)	Prayers and wishes n (%)	Seeking medical advice n (%)	Seeking medical/financial help n (%)	Offering medical/financial help n (%)	Analogy n (%)
General users, n=25,774	760 (2.9)^a^	1120 (4.3)^a^	613 (2.4)^a^	1864 (7.2)^a^	19,294 (74.9)^a^	260 (1.0)^a^	291 (1.1)^a^	35 (0.1)^a^	1537 (5.9)^a^
Patients with cancer/survivors, n=854	26 (3.0)	24 (2.8)^a^	464 (54.3)^a^	7 (0.8)^a^	64 (7.5)^a^	3 (8.5)^a^	183 (21.4)^a^	9 (1.1)^a^	4 (0.5)^a^
Relatives/friends of patients with cancer, n=1913	34 (1.8)^a^	42 (2.2)^a^	615 (32.1)^a^	16 (0.8)^a^	821 (42.9)^a^	137 (7.2)^a^	245 (12.8)^a^	0 (0)	3 (0.2)^a^
Cancer specialists, n=222	4 (1.8)	206 (92.8)^a^	5 (2.2)	0 (0)	0 (0)	4 (1.8)	2 (0.9)	1 (0.4)	0 (0)
Health-related accounts, n=459	46 (10.0)^a^	215 (46.8)^a^	46 (10.0)^a^	21 (4.6)	85 (18.5)^a^	13 (2.8)	20 (4.4)	7 (1.5)^a^	6 (1.3)^a^
Media-related accounts, n=680	62 (9.1)^a^	281 (41.3)^a^	104 (15.3)^a^	176 (25.9)^a^	24 (3.5)^a^	0 (0)	13 (1.9)	14 (2.1)^a^	6 (0.9)^a^

^a^Statistically significant at Bonferroni-adjusted *P*<.001.

## Discussion

The aim of this study was to assess the types of conversations and misconceptions regarding chemotherapy among Twitter users in Arabic-speaking populations. Given the large number of tweets retrieved, with their further spread via retweets, our study solidified the conclusions of previous studies that showed Twitter as a rich social media platform for obtaining health-related information [19-23]. Therefore, understanding these social media websites and having a glimpse of what is shared on these websites might serve as an important step for physicians to improve the health care delivered to their patients.

Twitter also represents a growing venue for researchers to analyze on what is shared about various topics of interest in health care either related to oncology [13-19] or related to topics on other health care fields (eg, antibiotics [24], vaccinations [25], smoking [26,27]). Twitter provides researchers and health care organizations with an opportunity to outreach a wide group of participants, overcome barriers pertaining to research resources, and track emerging health-related discussions and problems in real time [28,29].

In our manual content analysis, the most common theme of the tweets was “prayers and wishes.” This finding highlights a possible role of social media platforms as a support mechanism for patients with cancer receiving chemotherapy. Previous studies have also shown that social media platforms play a positive role in optimizing health care interventions [30], particularly in oncology settings [31].

Tweets containing misconceptions accounted for 7.0% (2084/29,904) of the total tweets in our sample population, making it the second most common theme. A further analysis of the misconceptions showed that most of these misconceptions were on the unrealistic side effects of chemotherapeutic agents, and people who do not have the knowledge or the experience of chemotherapy may tend to exaggerate their harmful effects, as reported in a previous study [5]. Therefore, health care organizations and professionals must tailor their awareness activities to target such common misconceptions in a given population.

Most of the tweets on media-related accounts delivered misconceptions about chemotherapy. We also found that when there was a spike in the misconceptions during the study period, the contributions of the cancer specialists and health-related accounts also increased proportionately to correct these misconceptions and to answer the questions of other users. This proportionate increase can limit the dissemination of misconceptions because cancer specialists and health-related accounts check the medical information that is tweeted and provide reliable information through tweets prior to sharing with the followers on Twitter. Further, heath care organizations can promote the true information shared by cancer specialists and health-related accounts by using the payable option offered by Twitter to show a certain tweet in search results and user feeds so that wide groups of the targeted population can be reached with reliable information [32].

Among the themes of the studied tweets, those containing prayers and wishes were the most common. This finding corroborates that reported in previous studies that showed that religious coping strategies were the most commonly followed strategies by patients with cancer [33]. A study on Arab women in Israel who had breast cancer showed that most of them used religious coping skills to actively cope with their disease [34].

This study had the following limitations. First, even though we searched for tweets containing representative spelling variations and abbreviations for the word “chemotherapy” in Arabic, there is still a possibility of some tweets being missed. For example, some tweets may have not mentioned chemotherapy directly but may have tweeted about it indirectly. Second, even with the large number of tweets retrieved and analyzed, the representativeness of the users and their age groups to the whole Arabic-speaking populations cannot be guaranteed. Third, the period of the study was 6 months. Longer study periods (eg, 1 year) will possibly give a broader view and include seasonal spikes such as special occasions such as Ramadan wherein prayers and wishes for recovery can increase, which might add valuable information to the current findings. Fourth, lacking automated content analysis might limit the reproducibility of the findings to some extent. Fifth, the generalizability of the findings is limited as tweets were subjected to multiple external factors that could not be controlled such as the timing of the tweets and the geographical place wherein the users lived [35]. Lastly, some users may have appeared multiple times within the data set which, to some extent, limits the interpretation of the statistical analysis, as their views on the matter being discussed might be overrepresented. Future studies should account for bots and assess for their contributions in conversations about chemotherapy on Twitter.

In conclusion, our findings corroborate those of the previous studies that showed Twitter as a valuable social media platform to assess public perception and misconceptions about various health-related topics. Most of the tweets in our sample population showed supportive messages for patients undergoing chemotherapy, thereby suggesting that Twitter could play a supportive role for such patients, while the second most prevalent theme of the tweets in our study was misconceptions about chemotherapy. The findings of our exploratory analysis can help physicians and health care organizations tailor educational efforts in the future to address the common areas of misconceptions about chemotherapy, thereby leading to increased public acceptance of chemotherapy as a suitable mode of treatment for cancer.

## Appendix

Multimedia Appendix 1 Arabic keywords used for searching tweets related to chemotherapy. All the keywords are the different possible spellings/abbreviations of the word "chemotherapy".
