# Supplementary material for: Conversations and Misconceptions About Chemotherapy in Arabic Tweets: Content Analysis
Source: J Med Internet Res. 2020 Jul 29;22(7):e13979. doi: 10.2196/13979 (PMC7424479; doi:10.2196/13979)
Supplement: Multimedia Appendix 1 [file jmir_v22i7e13979_app1.docx]

**Keywords used for searching for related tweet:**

1. الكيماوي
2. الكيميائي
3. كيماوي
4. كيميائي
5. كيمو
